# In Memoriam—Death of Horatio N. Loomis, M. D.

**Published:** 1881-04

**Authors:** 


### IN MEMORIAM.

### DEATH OF HORATIO N. LOOMIS, M. D.

The medical profession of Buffalo, and indeed of the State, have been called upon to mourn the death of one of its oldest and most honored members. At a meeting of the Erie County Medical Society, held at the Buffalo Medical College on Wednesday, March 23d, 1881, the following memorial, presented by Dr. Wyckoff, was unanimously adopted:

### MEMORIAL.

Horatio N. Loomis, for forty-five years a resident and practitioner of medicine in this city, died of pneumonia, after an illness of four days at his residence on the corner of Frrnklin and Niagara streets, on Tuesday, March 22d, 1881, at the age of seventy-four years. Dr. Loomis was born at Franklin, New London county, Conn., on March 25, 1807. He moved with his father to Saugersfield, Oneida county, N. Y., when a lad of five years of age. His preliminary education was obtained at the schools and academy of the neighborhood where he resided. He attended three or four courses of medical lectures, and graduated at the college of Fairfield, Herkimer county, N. Y., in the year 1828. After having practiced his profession for a few years at Palmyra, in this State, he removed with his family to Buffalo in the year 1836, where he had continually practiced his profession up almost to the day of his death. He had been a member of the Erie County Medical Society forty-five years, and at the time of his death was its oldest member.

The records of this Society should bear testimony to the valuable services of those who go without reward often, save the consciousness of duty performed. This Society desires to express its testimony to the marked professional ability and untiring devotion of Dr. Loomis to the arduous duties of the medical profession during a period of more than half a century. The removal of such a man in the fullness of his years and usefulness from the presence of his friends and the sphere of his daily duties; of one so gentle and loving in the home circle; so genial and kindly among his associates; so conscientious and faithful in professional responsibility; so upright and honorable as a citizen, claims from us what we most heartily accord, a generous acknowledgement of his professional worth; our warmly cherished respect for his memory, and our abiding sympathy with his family in the great bereavement to which they have been called.

*Whereas*, God in his all-wise providence has been pleased to remove from the scene of his earthly labors our revered and honored professional brother, Dr. Horatio N. Loomis, and since it is becoming and proper that we who have known the deceased so long and under circumstances so favorable to a just appreciation of his moral and professional worth, should give expression to the respect we cherish for his memory; therefore be it

*Resolved*, That in the death of Dr. Horatio N. Loomis, the medical profession of Buffalo have lost one of their leading members, the community one of its most useful citizens, who was universally esteemed and admired for his sound, practical wisdom and professional attainments.

*Resolved*, That Dr. Loomis, in his entire and exclusive devotion to his profession' and his diligence in the discharge of its duties during a long lifetime, to the last keeping pace with it in its steady advancement, and in his scrupulous observance of every professional courtesy to his patients and to his medical brethren, has left behind him an example of dignity, fidelity and success, which may be held up to the profession as worthy of imitation.

*Resolved*, That we deeply sympathize with the family of the deceased in a loss which to them is truly irreparable.

*Resolved*, That we will, as a society, attend the funeral of Dr. Loomis in a body.

